# Advances in Directly Amplifying Nucleic Acids from Complex Samples

**DOI:** 10.3390/bios9040117

**Published:** 2019-09-30

**Authors:** Faye M. Walker, Kuangwen Hsieh

**Affiliations:** 1 Raytheon Company, Waltham, MA 02451, USA; faye.m.walker@raytheon.com; 2 Department of Mechanical Engineering, Johns Hopkins University, Baltimore, MD 21218, USA

**Keywords:** nucleic acid testing, sample preparation, disease diagnostics, bubble plot

## Abstract

Advances in nucleic acid amplification technologies have revolutionized diagnostics for systemic, inherited, and infectious diseases. Current assays and platforms, however, often require lengthy experimental procedures and multiple instruments to remove contaminants and inhibitors from clinically-relevant, complex samples. This requirement of sample preparation has been a bottleneck for using nucleic acid amplification tests (NAATs) at the point of care (POC), though advances in “lab-on-chip” platforms that integrate sample preparation and NAATs have made great strides in this space. Alternatively, direct NAATs—techniques that minimize or even bypass sample preparation—present promising strategies for developing POC diagnostic tools for analyzing real-world samples. In this review, we discuss the current status of direct NAATs. Specifically, we surveyed potential testing systems published from 1989 to 2017, and analyzed their performances in terms of robustness, sensitivity, clinical relevance, and suitability for POC diagnostics. We introduce bubble plots to facilitate our analysis, as bubble plots enable effective visualization of the performances of these direct NAATs. Through our review, we hope to initiate an in-depth examination of direct NAATs and their potential for realizing POC diagnostics, and ultimately transformative technologies that can further enhance healthcare.

## 1. Introduction

**Figure 1:**
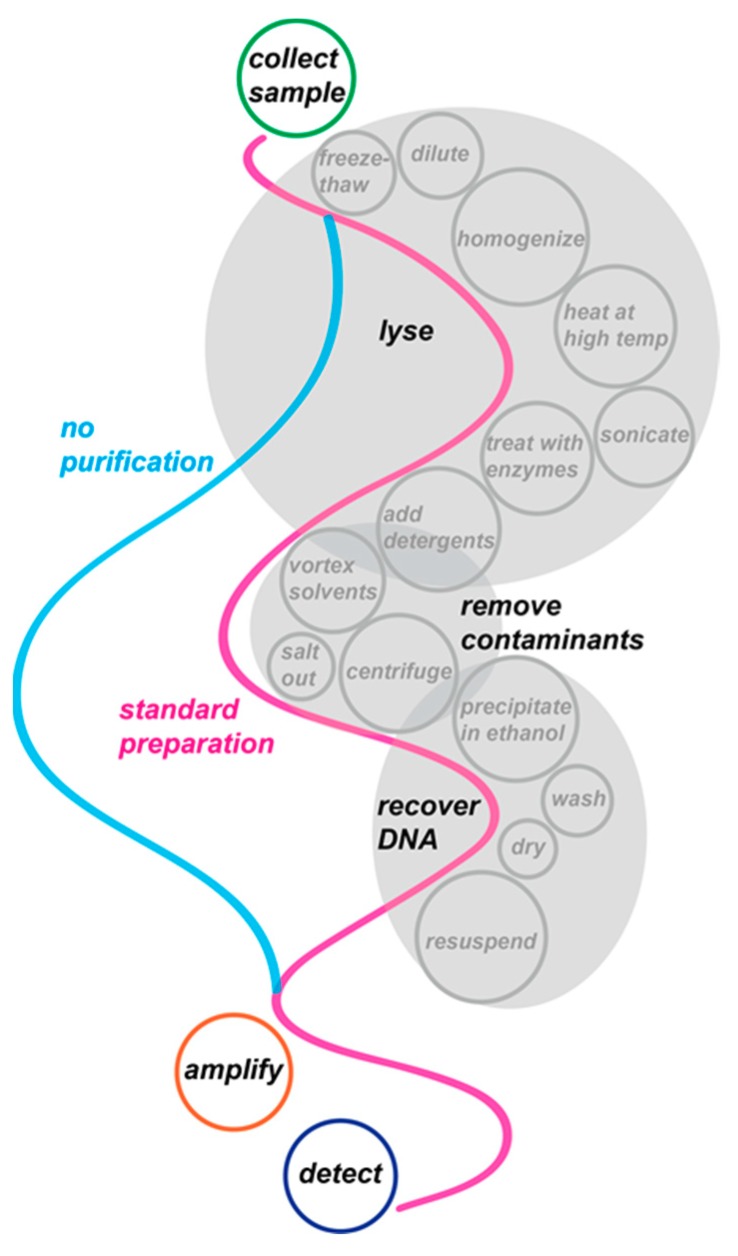
Direct nucleic acid testing is much more convenient and streamlined than the three-step method with preparatory techniques. In a typical extraction experiment, buffer with lytic agents is added to dilute the sample and homogenized with a mixer. Sonication creates pressure waves that burst the cells in mechanical lysis. Lysozyme enzymatically destroys cells, and is removed from the reaction with vortexing and centrifugation in a phenol/chloroform phase separation. The DNA is precipitated in fresh ethanol and the resulting mixture is washed to remove excess contaminants. Excess liquid is removed so that the DNA can be resuspended in an appropriate buffer.

Nucleic acid amplification tests (NAATs) have become indispensable tools in biology and medicine. For example, for infectious diseases diagnostics, NAATs are generally faster, more sensitive, and more specific than the current gold standard of culture-based techniques. In fact, a number of DNA- and RNA-based diagnostics are now recommended by the US Food and Drug Administration (FDA) for infectious diseases such as human immunodeficiency virus (HIV) [1,2]. Bringing NAATs to the point of care (POC), and particularly to resource-poor settings, is envisioned to revolutionize healthcare. Unfortunately, many NAATs require access to expensive, specialized equipment and a degree of expertise that is highly unlikely to be found in decentralized laboratories. As an additional challenge, these tests typically require an extraction step to isolate DNA or RNA from blood, urine or sputum, and a purification step to eliminate contaminants from the sample matrix that can confound the actual detection procedure (Figure 1). These procedures necessitate expensive instrumentation and can add up to several hours to sample-to-answer results, which further restricts the use of NAATs within centralized laboratories.

Many groups have attempted to develop portable, integrated, microfluidics-based platforms to increase the functionality of diagnostic sensing and analysis [3,4,5], and some of these have even been commercialized (e.g., bioMerieux’s NucliSENS easyQ tests, TwistDx’s TwistAmp kits, and Enigma Diagnostic’s MiniLab). These platforms present breakthrough technologies for rapid, cost-effective, and user-friendly diagnostics. While it remains to be seen whether these systems are simple and error-free enough for developed and developing settings, they demonstrate the feasibility of implementing existing nucleic acid amplification methods for POC use [6,7,8,9].

An alternative approach to time-consuming and cumbersome sample preparation is performing NAATs directly from complex samples (Figure 1). The advantage of traditional amplification technologies, such as PCR with real-time spectroscopic or mass spectrometry detection, is that the results are highly specific and quantitative. However, these sensing platforms are expensive and require prior extraction of genetic material from the sample.

Direct NAATs are advantageous when complicated, costly laboratory apparatuses are not available. They not only reduce the time, labor, and technical constraints of molecular testing, but also bring the additional benefit of standardizing results [10]. Indeed, a growing number of groups are developing such “direct” NAATs. Most notably, the Alere i Influenza A&B assay became the first FDA Clinical Laboratory Improvement Amendments (CLIA)-waived nucleic acid-based test [11] in January 2015. As the Alere i system requires no front-end nucleic acid extraction, and can be used outside of traditional laboratory sites [12,13,14,15,16], its development and CLIA-waived status provide strong support for further development of direct assays that can minimize or even bypass sample preparation.

Thus motivated, we present the current state of direct assays and platforms that achieve nucleic acids detection and analysis from clinically-relevant, complex samples but with either minimal or even no sample preparation procedures. We surveyed the literature from 1989–2017 and came across a significant number of works that reported NAATs from bodily samples (e.g., blood-based liquids, oral samples, swabs) without the complex steps generally involved in sample preparation. This meant discarding the works that depended on sophisticated instruments and operations that are labor-, time-, and cost-intensive, such as enzymatic (proteinases), chemical (acids, detergents), or physical (temperature shock, mechanical disruptions) treatments. Then, we describe examples whereby data visualization can be used to reveal the connections between the robustness, sensitivity, and efficacy of technologies developed for direct DNA- and RNA-based tests. It is our hope that in reviewing technologies such as these, and presenting these promising early findings in an information-rich and accessible fashion, we can help to accelerate the development of approaches that make POC nucleic acid testing rapid, accurate, simple, and affordable.

## 2. Methods

In order to find relevant articles with data on NAAT parameters, we performed literature searches from December 2014 to February 2018.

### 2.1. Literature Search

We searched Google Scholar with a combination of search terms. These followed a formula of combining a descriptor (e.g., “point-of-care”), an amplification technology (e.g., “LAMP” OR “loop-mediated isothermal amplification”) and a sample matrix (e.g., “blood”). References of previously published reviews, as well as those included in original studies, were checked for possible candidate articles.

### 2.2. Record Screening

Articles were initially screened on the title, and secondly on the abstract. Any articles that relied on microfluidic platforms or commercialized extraction devices were excluded. Publications that required complex pre-processing with enzymatic treatment or chemical purification were not selected. Studies were included if they involved direct amplification and detection of genetic material from one of six representative sample types: blood, dried blood spot, serum and plasma, saliva and sputum, swabs, urine, and stool. The full text of appropriate articles was read to extract the necessary information.

### 2.3. Data Abstraction

From each of the 174 published works surveyed, we extracted and recorded data that corresponded to test performance. There are many parameters that cannot be ignored when considering NAATs: accuracy, specificity, user-friendliness, training requirements, and so on. As such, we provide an extensive examination of nucleic acid template specificity (including single or multiplexed reactions), amplification methodologies (enzymes, operating temperatures, and amplification technology), and user-friendliness (storage considerations, pretreatment requirements, and physical involvement) in Supplementary Table S1.

In addition, we have classified assays that can feasibly be completed without extensive training or high-end instrumentation as “direct,” whereas those with greater labor or equipment demands (e.g., freezers, high-speed centrifugation, or incubation for multiple hours) are deemed “semi-direct.” Specifically, the “semi-direct” assays have the following exceptions to a simple laboratory setup: alternating between two or more incubation temperatures (other than room temperature), relying on enzymatic activity, or requiring more than a brief, low-speed (<100× *g*) centrifugation. Methods categorized as “semi-direct” face some hurdles to implementation as an on-site service for patient care. What these tests do offer is a way to deliver actionable results that can link diagnosis to treatment. With appropriate conversion from requirements for highly trained staff and sophisticated tools to easy-to-use methods, “semi-direct” procedures will meet the requirements for POC diagnostic devices.

### 2.4. Visualization Process

We found certain parameters could be distilled into numerical data, yielding particularly useful insights when examining different tests. We have devised three major criteria that are indicative of each platform’s robustness, sensitivity, and clinical efficacy:

1. Tolerance to the sample of interest—ideally, the assay should be able to detect its target against a high concentration of background contaminants. We note that, although sample dilution sometimes provides a convenient way of permitting amplification, doing so inevitably reduces the limit of detection (LOD). Most NAATs analyze only a fraction of the sample volume. Sample dilution therefore increases the likelihood of false negative results, especially when the samples already have low target concentrations.
2. LOD—by foregoing sample preparation, one generally sacrifices the opportunity to concentrate bulk samples, reducing the limit of detection and making sensitivity an important consideration.
3. Clinical evaluation—recognizing assays that have been validated with clinical samples.

Finally, we sought to devise a visual strategy that would clearly and quickly communicate the importance of our criteria, compare the wide range of assays, discover trends in the data, and reveal patterns in a single glance. Specifically, the essential information of the 174 reviewed publications is presented quantitatively in a single plot. Relevant values are standardized and communicated in terms of visual attributes of position, size, shape, and color.

We have found it particularly useful to visualize the data as “bubble plots.” In a bubble plot, numerical values from three parameters are simultaneously visualized via the two axes and the size of the circular marker. Different categories can also be grouped according to the color of the markers. In our case, we can readily display the essential information (e.g., sample tolerance, LOD, and instances of clinical testing) of related procedures to discern those that enhance test performance.

## 3. Brief Overview of Isothermal Amplification Techniques

In our survey, we came across eight DNA- and RNA-based testing techniques. As expected, PCR (and reverse transcription PCR, or RT-PCR) has been the predominant technique. Notably, a number of isothermal amplification techniques have also been used to develop direct NAATs. Herein, we provide brief overviews of these lesser known isothermal amplification techniques.

### 3.1. LAMP

While PCR is the most commonly reported method of amplification, there is an increasing number of isothermal amplification technologies that can be truly used at the POC. The single reaction temperature enables the use of less costly, complicated instruments than for thermal cycling tests. Loop-mediated isothermal amplification (LAMP) is one such widely researched, developed, and characterized method [17]. Amplification employs a strand-displacing polymerase and two or three pairs of primers: one that is sacrificed to linearize the template, and one or two others that prime the DNA synthesis to produce concatenated, cauliflower-like products [18]. As with PCR, LAMP has been modified to target RNA as reverse-transcription (RT)-LAMP [19].

LAMP has been compared to PCR in other ways as well, including applications with bacterial, viral, fungal, and parasitic assays. Not only has the specificity and sensitivity been equivalent to that of PCR, the robustness of LAMP to certain preparations of serum, swabs, and blood has shown it to be more tolerant to inhibitors than PCR [8].

### 3.2. NASBA

The nucleic acid sequence-based amplification (NASBA) method is unique in its ability to amplify single-stranded RNA directly [20]. This is most desirable for targeting RNA viruses and for transcriptome analysis [8]. The continuous, homogeneous, isothermal process relies on RNA polymerase, RNase, and reverse transcriptase. First, the reverse transcriptase creates a double stranded RNA:DNA hybrid from the RNA template; next, the original RNA is destroyed; a DNA duplex is synthesized; then, the polymerase can transcribe RNA from the DNA. Each new RNA molecule can repeat the cycle for exponential amplification.

NASBA has been applied to a wide-ranging set of research problems, including HIV diagnosis during the AIDS epidemic of the 1990s and automated, real-time, clinical tests in blood with the modern NucliSENS (bioMerieux, Inc., Durham, NC, USA) or in urine with the APTIMA assay (Hologic, San Diego, CA, USA). NASBA is also used outside of the commercial sector with systems to monitor viruses in serum [21].

### 3.3. SDA

The strand displacement amplification (SDA) technique is based upon the abilities of a restriction enzyme and a DNA polymerase. A primer containing a recognition sequence for the restriction enzyme binds to its complementary, single stranded DNA target. After extension by the polymerase, the restriction enzyme nicks the unmodified strand of the double-stranded hemiphosphorothioate recognition site. DNA polymerase then extends the 3′ end of the nick, displacing the downstream strand. The end result is exponential target amplification from the displaced strands, which serve as targets for new reactions.

SDA is not complex, but it does suffer from sensitivity issues in the presence of background DNA. The best way to overcome off-target amplification, and hence reduce false-positives, is to use simple pretreatment procedures like those that have been developed for detection with the BDProbe-Tec (Becton Dickinson Microbiology Systems, Sparks, MD, USA) and in-house systems for urine [22].

### 3.4. RPA

Recombinase polymerase amplification (RPA) avoids thermal cycling by using three core proteins that operate optimally between 37–40 °C [23]. The first protein, recombinase, binds to primers that recombine with a duplex target for strand displacement. The second, a single-stranded DNA binding protein, attaches to the displaced strand before a strand-displacing polymerase copies the DNA from the primer onwards for exponential amplification.

One of the requirements for RPA technology is sequence-specific detection. This avoids the problem of primer artifacts that add to background fluorescence with nonspecific, intercalating dyes. With its specific readout and rapidity (<20 min to results) as two main features, RPA provides an alternative to the time-consuming processes of culturing and bacterial genotyping when testing for pathogens [7].

### 3.5. SIBA

Strand invasion based amplification (SIBA) is another amplification process that relies on recombinase activity. In SIBA, there is a separate recombinase substrate that is inserted between two primer-binding sites. The duplex peripheral to this insertion site is separated, enabling the primers to bind. DNA polymerase can then extend the template from the bound primers. This use of an invading substrate, one that is neither consumed nor included in the extension of DNA, is advantageous because it abolishes primer artifacts. SIBA can therefore be used to reliably detect low copy numbers of pathogens—other isothermal methods generate non-specific amplification products in the absence of target DNA [24]. Going further, the specificity of SIBA enables multiplexing for the detection of templates that differ by as little as two bases [25].

### 3.6. MDA

Multiple displacement amplification (MDA) is a technique that exploits the strand displacement, proofreading, and polymerase activity of the ϕ29 bacteriophage DNA polymerase [26]. The highly processive polymerase uses random primers to amplify an entire genome. MDA is therefore well-suited for whole genome amplification from crude biological samples, which can be followed by single nucleotide polymorphism (SNP) testing and genotyping [8].

### 3.7. HCR

The concept of hybridization chain reaction (HCR) [27]—an enzyme-free, room-temperature method—relies on a DNA trigger to initiate amplification. The initiator interacts with two stable DNA hairpins to create nicked double helices. Amplification of this initiation event continues until the hairpins are depleted. HCR is a useful assay for detecting short DNAs, such as human immunodeficiency virus type 1 (HIV-1) in serum [28].

## 4. Analysis of Surveyed Direct NAATs

### 4.1. Growing Prevalence of Direct NAATs 

**Figure 2:**
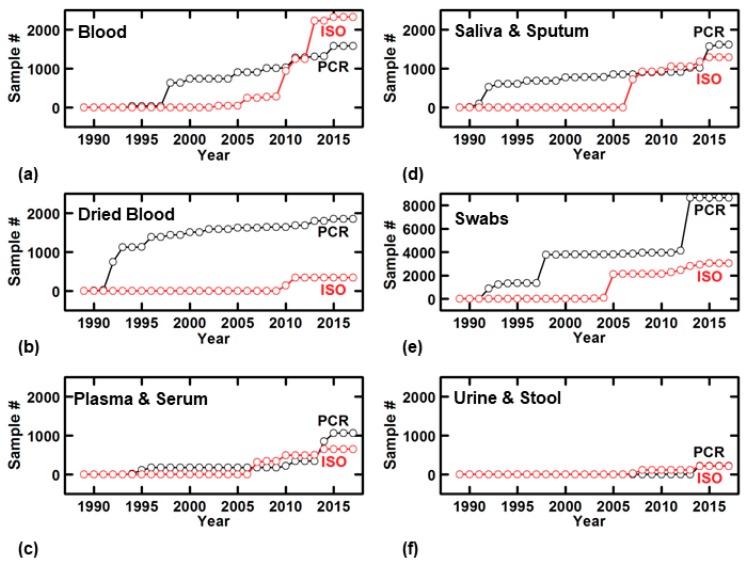
Time-series data for direct nucleic acid diagnostics according to accrued number of samples tested. Individual plots of number of clinical samples over time are subdivided according to sample matrix as follows: (**a**) whole blood, (**b**) dried blood, (**c**) plasma and serum, (**d**) saliva and sputum, (**e**) oral, dermal, and conjunctival swabs, and (**f**) urine and stool.

Time-series plots offer an effective means for showing the growing prevalence of direct NAATs. Specifically, within each sample type, we plotted the number of clinical samples that have been analyzed by direct NAATs from 1989 to 2017 (Figure 2). Here, we also divided the data into two cohorts based on whether samples were subjected to PCR (Figure 2, black) or isothermal amplification techniques (Figure 2, red). As expected, we saw an overall rise in the number of clinical samples analyzed via NAATs with minimal or no sample preparation over the analyzed period. Across all six sample types, we observed sharp spikes, which indicate studies of high numbers of clinical samples. Based on the sample type, swab samples had been most analyzed, while urine and stool samples had been least analyzed. Within each sample type, after an initial lag, we saw a notable rise in the use of isothermal amplification techniques. This first became apparent as early as 2003, several years after the advent of LAMP in 2000, and twelve years after the introduction of NASBA [20]. PCR-based systems emerged within five years of the technique’s inception in 1986 [29], and PCR largely continues to dominate the realm of nucleic acid testing. The one notable exception is seen in our time-course of blood testing, where isothermal techniques have surpassed PCR and RT-PCR in terms of the number of assays performed on whole blood samples.

### 4.2. Direct NAATs for Whole Blood

Because blood contains circulating nucleic acids, cells, and over 20,000 different proteins, it offers an abundance of biomarkers for disease detection. Molecular diagnostics in blood are useful for detecting specific DNA or RNA sequences from a range of bacterial, toxic, and viral infectious agents. Platforms for hepatitis and human immunodeficiency virus [30], *Staphylococcus aureus* [31], and *Plasmodium* species are just a few of the most-used systems enabling rapid diagnostics in whole blood.

Blood-based testing generally demands sophisticated detection instruments or extensive preparation to recover inhibitor-free and high-purity DNA. Not all inhibitory blood components are known [32], but heme compounds, anticoagulants, and immunoglobulin G (IgG) can all interfere with amplification reactions by inhibiting DNA polymerase activity [33] or chelating necessary cofactors [34,35]. Although a wide range of bloodborne viruses, bacteria, and parasites can in principle be detected with nucleic acid testing, extraction- and purification-free means of detecting these pathogens are not currently commercially available.

**Figure 3:**
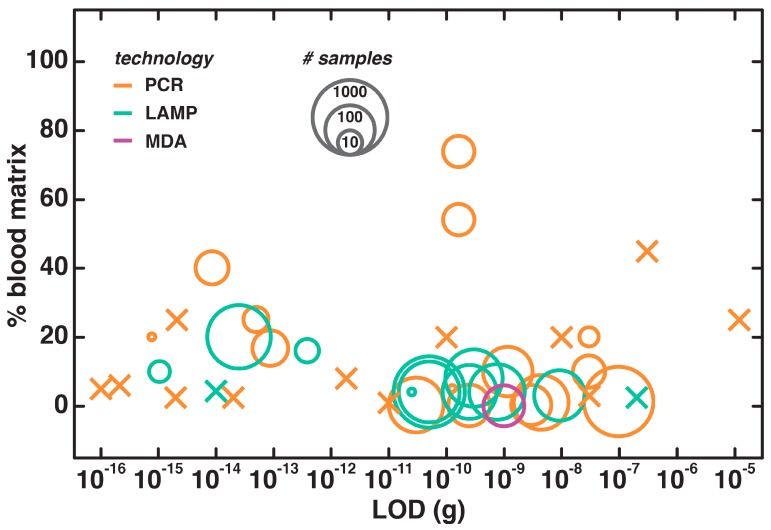
Bubble plot of nucleic acid diagnostics performed in whole blood. Percent concentration (*v*/*v*) of blood per reaction in a given procedure is displayed as a function of the limit of detection (LOD) in g of template. The number of patient samples tested is proportional to the log of the marker area, as shown at top, and the testing methodology is indicated by marker color. Cases shown with × instead of bubble markers illustrate that patient testing was not reported.

We have visualized the general trends of direct and semi-direct nucleic acid testing in blood as a function of the LODs (Figure 3). The % (*v*/*v*) of blood tolerated in a reaction is plotted against the LOD in g of template, with the number of clinical samples encoded as the area of the bubble. We have also assigned colors to indicate the type of amplification technology. It is evident that many studies have achieved high sensitivity in detecting their target in a low concentration of blood. This shows that nucleic acid testing has great potential for blood-based tests in POC situations where collection volumes are small (e.g., finger pricks) and parasite loads may be low. Those examples from the literature that were not demonstrated on patient samples are considered separately in the plot, and represented by Xs rather than bubble markers. Some of these are purported to have very low LODs that reach below the fg level (Table S1)—it remains to be seen whether such tests will perform with the same extreme sensitivity in a clinical context.

By assessing the PCR- and isothermal-based data, we could obtain some insight into how to optimize these techniques to better tolerate blood as a sample matrix. Several of the semi-direct works with PCR have employed over 50% blood in a reaction after heat-cold shock [36]. More noteworthy is a truly direct example that relied on the specificity and efficacy of the Phusion polymerase (New England Biolab, Ipswich, MA, USA) to perform PCR in 40% blood [37]. PCR typically employs the Taq polymerase from *Thermus aquaticus*. Chemical additives, whether commercially-available cocktails [38,39] or in-house buffers [39,40,41,42,43,44,45,46,47,48,49,50,51,52], allow the Taq family of polymerases to amplify DNA from whole blood. PCR can likewise be optimized through the use of more unconventional polymerases [53,54,55,56,57,58] and physical heating steps [53,59,60,61,62,63,64] to reduce the inhibitory effect of blood components. These referenced works offer expedited methods to obtain amplifiable templates with similar sensitivities to chemical-based extraction kits [59]. Though several authors include the use of a centrifuge in the extraction process, these semi-direct methods of template preparation could likely be completed by relying on careful pipette-based transfer of supernatants rather than high-speed centrifugation [59,63].

Most isothermal amplification-based diagnostics in blood make use of LAMP [17], which offers a highly tolerant means of amplification [8]. Simple treatments with heat [59,65,66,67,68,69,70,71,72,73,74,75,76] or chemicals [73,77,78,79,80,81] can increase the sensitivity of the LAMP or RT-LAMP reaction. Most impressive are the examples of direct amplification of DNA in blood with LAMP-based technologies [82,83] and other isothermal amplification methods like MDA [84]. Some of these assays employ a post-heating centrifugation step, but since Poon et al. have demonstrated that LAMP can be performed directly on heat-treated blood without a spin-down process, this step could likely be avoided in semi-direct processes [68].

Even though these successful examples of simple, direct nucleic acid testing methods highlight the promise of DNA amplification in whole blood, there is an ongoing need for further improvements. No assay has come close to reaching the capacity of Burckhardt et al.’s PCR amplification with Taq polymerase in nearly 80% whole blood, as demonstrated over 20 years ago in 1994 [36]. The associated treatment method is one of the more technically-involved and time-intensive, demanding 20 cycles of heating and cooling. It remains to be seen whether an isothermal amplification method could equal this tolerance. Perhaps these techniques will make up for their decreased level of tolerance in their ease of use, as evidenced by Suzuki et al. achieving 20% incorporation of whole blood in LAMP with only a five-minute heating [76].

### 4.3. Direct NAATs for Dried Blood 

Dried blood spots offer a convenient alternative for screening for genetic disorders, testing for infectious diseases, and profiling drug metabolism in settings with limited laboratory or storage capabilities. Such samples are typically prepared by spotting whole blood, either from venous blood or a finger prick, onto filter paper [85]. Sampling time is quick, temperature-controlled storage is unnecessary, and biohazard risks are minimized for health care workers [86]. The downside of such samples is that the DNA in the dried blood must be eluted from the paper-based cellular components before it can be amplifiable. 

Filter paper has been used as medium to test blood for infectious diseases since the 1940s [87]. From syphilis diagnosis during World War II [88], to infant screening in the 1960s [89], to HIV detection and monitoring in the modern day [85,90], there are important assays with dried blood spots in NAATs. Commercial technologies are even becoming widely available to map, monitor, and survey blood spots from patients infected with malaria or other neglected tropical diseases [74,91]. In a similar manner, the preparation and processing techniques for dried blood samples presented below could open new avenues for disease control and elimination when combined with well-standardized assays for detecting bloodborne pathogens.

**Figure 4:**
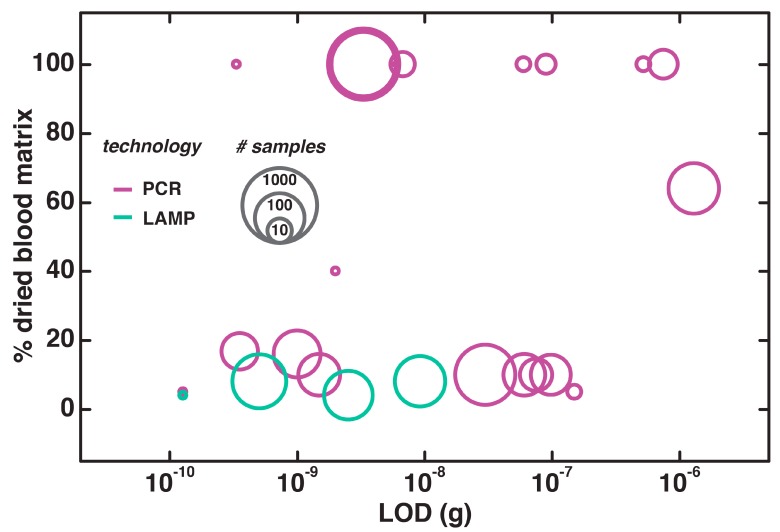
Nucleic acid testing with dried blood spots, with data presented as in Figure 3.

As shown in Figure 4, all of the tests we surveyed have been validated with actual dried blood spots. In the most-heavily tested example (720 clinical samples) by Raskin et al., pretreatment with heating-cooling cycles and addition of spermidine to the reaction helped boost the efficiency and yield of the amplification [92]. Blood spot-containing filter paper can typically be directly added into a mixture of reagents, though it necessitates overcoming the high background interference of the filter paper and detecting small amounts of dried blood.

To overcome the background interference from filter paper in directly amplifying dried blood spots via PCR, researchers have bolstered the enzyme’s resistance to inhibitors and included various buffer components [47,93]. Pretreatments, such as fixing [92,94,95,96,97] or heating [43,98,99,100], also aid in improving sensitivity and specificity. Even if the procedures are reported as being too lengthy for POC, there are appropriate ways to scale down the waiting period: for instance, an overnight drying period with methanol while under vacuum [98] can be streamlined into a five-minute methanol fix [100]. Most of the approaches to amplify DNA directly in blood spots use eluants—either from commercial kits [51,101], in-house buffers [44,59,102,103,104,105], or water [106,107]—to overcome the various difficulties that impede PCR reactions with paper matrices. Buffer-based eluants, in addition to being cost-effective, can achieve even higher sensitivities than standard extraction protocols [105]. Similar preparatory approaches are used for LAMP, wherein heating in water [72,108], phosphate buffered saline (PBS) [59], or sodium dodecyl sulfate (SDS) buffer [74] enables a fast and easy nucleic acid elution with amplification results that are comparable to the conventional gold standard of microscopy [72,74,108].

One of the difficulties in making blood spots suitable for LAMP and PCR is the need to re-suspend the spots in liquid, then filter out the species of interest. The easiest way to address the former problem is through long elution times; the latter, through centrifugation. This makes Taylor et al.’s amplification of *Plasmodium* spp. DNA directly from clinical filter paper samples such a remarkable achievement for low-resource settings. The combination of an inhibitor-resistant Taq mutant and an enhancer cocktail resulted in a specificity and sensitivity of 100% for 48 patient samples [47]. All the approaches have interesting characteristics that make them special, but none achieve the ease in use of this assay for malaria.

### 4.4. Direct NAATs for Plasma and Serum

Blood plasma and serum are widely used for quantitative molecular diagnostics in the areas of clinical decision-making and therapeutic management [109]. Plasma is the pale yellowish fluid that normally holds the blood cells of whole blood in suspension, whereas serum is the remnants of blood plasma after the removal of clotting factors [110]. Circulating DNA in serum and plasma is a biomarker for a diverse array of systemic, infectious, and genetic diseases. These include particular disorders such as diabetes [109] and hepatitis B virus [111].

Refining blood into serum or plasma historically requires expensive equipment for centrifugation or sedimentation. Recovering DNA or RNA from blood-based proteins, nutrients, electrolytes, antibodies (particularly IgG), antigens, hormones, and exogenous substances becomes even more challenging when considering the low relative levels of cell-free or cell-bound nucleic acids [112,113,114]. More recently, however, paper- or card-based devices [115,116], membrane-based sedimentation [117], and microscale devices for cell differentiation and filtration [118] have made blood separation a single step process at the POC. As such, we include these sample types here.

**Figure 5:**
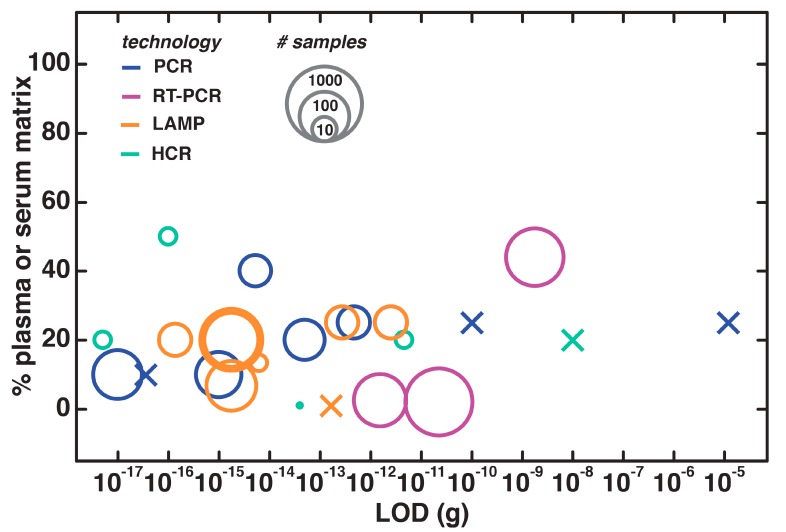
Serum and plasma-based nucleic acid testing, with data presented as in Figure 3.

In assessing nucleic acid testing with plasma or serum, we see that most reactions are performed at sample concentrations in the 20% range (Figure 5). However, it is important to note that the sensitivity does not necessarily suffer in much more concentrated samples—in Liu et al.’s highly robust two-step amplification process with direct hairpin assembly and HCR-based detection of SNP DNA sequences in 50% (*v*/*v*) serum, they achieved a very low LOD of 100 pg [119]. These plasma/serum-based tests are especially promising for use in real-world contexts, because their clinical relevance is well-documented—18 out of the 24 cases examined here included testing with patient samples.

Researchers have developed convenient PCR assays for both direct and semi-direct testing in blood-based fluids. By using enhanced enzymes, DNA [49,50,67,120] and RNA [121,122] targets have been successfully amplified in plasma and serum. Additionally, heat-based pretreatment can be used to release nucleic acids prior to carrying out amplification [36,60,123,124,125,126].

The effect of preheating can be seen in LAMP as well. LAMP is generally tolerant to the serum or plasma environment [120,127], but preheating the input sample has been found to have a favorable effect [128,129,130,131] that produces up to a 100-fold improvement in sensitivity [132]. This heating also enabled Pardee et al. to detect Zika virus RNA in serum with high sensitivity using NASBA [21]. HCR performs especially well in serum without any pretreatment [28,119,133,134,135], presumably because the reaction relies on cascaded hybridization events instead of polymerases.

Because plasma and serum contain very low-abundance analytes, nucleic acid tests need to operate with high sensitivity. Fortunately, LAMP-based applications are achieving increasingly low limits of detection. Nijru et al., for instance, demonstrated that their LOD of 1 *Trypanozoan* parasite/L serum in HAT diagnosis was 100-fold more sensitive than PCR testing. Such methods could still benefit from user-friendly techniques for large-scale processing. Some semi-direct examples presented above include a centrifugation step to collect condensate formed after heating, but could just as easily rely on pipette collection to obviate the need for a high-speed centrifuge. Others might benefit from certain stand-alone modules for plasma and serum separation that could be integrated into a POC workflow [117,136].

### 4.5. Direct NAATs for Saliva and Sputum

Saliva and sputum are abundant and easy to obtain, and are thus attractive samples for diagnostics. Saliva flows into the oral cavities through salivary glands, where blood vessels secrete the same protein and nucleic acid biomarkers as in peripheral blood. In contrast with blood-based samples, saliva sampling does not require trained technicians, presents fewer antigen-associated risks, and can be more easily purified (saliva is 95% water) [137]. Sputum, a necessary sample for respiratory infections, is mucus from the lower airways. Unfortunately, saliva and sputum are very heterogeneous with respect to the distribution of organisms, chemical composition, and the presence of outside contaminants such as toothpaste, cigarette smoke, coffee, or mouthwash. Technical extraction kits such as RNaqueous and MagMAX (Life Technologies, Grand Island, NY, USA) are often used to eliminate inhibitors and nucleases from oral samples. The viscosity of sputum requires particularly cumbersome protocols for sample preparation: full processing begins with mucolytic agents such as *N*-acetyl-*L*-cysteine (NALC) and dithiothreitol (DTT), disruption of mycobacteria by detergents and proteolytic enzymes, then isolation of target DNA by organic solvents or capture reagents [138,139].

The human salivary microbiome has importance as a diagnostic indicator of oral cancer, oral diseases such as periodontitis, and systemic diseases such as pneumonia [140]. As for sputum, it has become the specimen of choice for detecting tuberculosis [141,142]. NAATs for *Mycobacterium tuberculosis* have been endorsed by the WHO (World Health Organization) and the FDA for their high accuracy [143,144]. The systems introduced below extend the practical usage of sputum for POC testing by reducing the requirements for sputum manipulation. 

**Figure 6:**
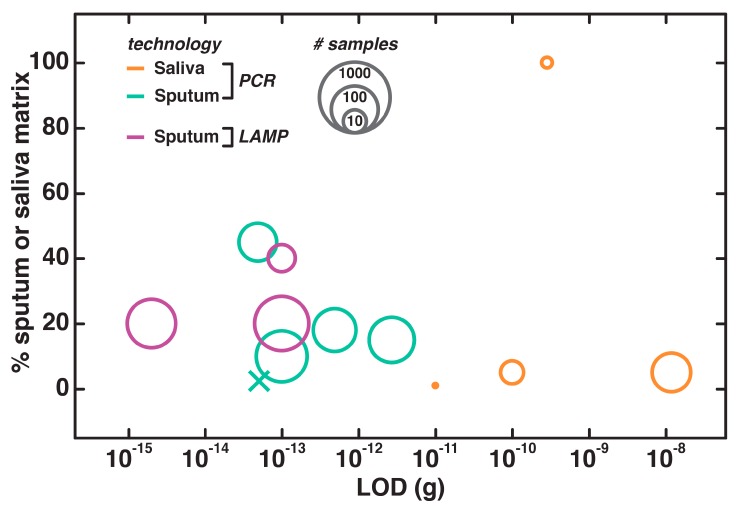
Nucleic acid testing procedures in saliva and sputum, with data presented as in Figure 3.

Most reported nucleic acid testing methods for saliva and sputum show fairly high numbers for patient samples tested, with only two of 30 cases that did not examine clinical specimens (Figure 6). The approaches we examined generally employ dilutions of 20% or less, and achieve low detection limits. This is critical for avoiding false positives, as the target concentrations in sputum and saliva are small. The lowest LOD achieved—2 fg of *Acinetobacter baumannii* bacterial gDNA in sputum—required pretreatment with Sputazyme (Kyokuto, Tokyo, Japan) and heat before LAMP analysis [145]. In contrast with blood, the high water content of saliva should make it relatively easy to augment the concentration of matrix that can be employed in an amplification reaction. There are several examples of amplification directly on dried sputum collected via filter cards, which is an especially promising direction for direct testing at the POC if samples need to be stored, handled by multiple clinicians, or reevaluated at later dates [87,146,147].

Amongst the collection of approaches for direct PCR amplification on saliva samples, those that begin with dried saliva swabs fully circumvent DNA extraction, purification, and quantification [93]. Hall et al. added punches of saliva stains directly to the reaction mixture in order to perform STR analysis. Genetic testing in saliva or sputum is often performed directly in liquid samples, and dilution into the reagents is typically sufficient to negate the effect of any inhibitors [148,149,150], although heat [151] or additives such as polyethylene glycol (PEG), hydroxides, or dithiothreitol (DTT) may be added to further process samples [42,43,56,145]. With the broad range of bacterial species present in the mouth (over 600 inventoried), differing in terms of their contributions to health and disease, PCR is very useful for profiling large numbers of bacteria. Some species are recalcitrant to lysing, like *Streptococcus*. So, instead of relying on bead-beating, phenol treatments, or other steps, Aas et al. applied proteinase K lysates directly to PCR reagents [140]. Several other groups have followed suit in detecting bacterial taxa in healthy [152,153] and diseased saliva samples [154]. Direct amplification is also possible in the field of LAMP-based diagnostics, as evidenced in testing for Zika [82] and malaria [65]. Du et al. went further than simplifying the sample treatment, a ten-minute heating of Zaire Ebolavirus DNA in saliva, by actually providing a LAMP-to-glucose transduction that can be read out on a handheld glucometer [155].

Designing direct tests for sputum is inherently difficult because many nucleic acid-based methods process samples analogously to culture-based protocols. In this *N*-acetyl-L-cysteine (NALC)-NaOH method [156], the viscous sputum matrix is liquefied through several buffer exchanges and high-speed centrifugation into a more manipulative sample for testing. An additional concern is decontamination. This is necessary for culture, but also useful to protect the operator from biosafety hazards in molecular testing [157]. Several semi-direct examples based on LAMP [158,159,160], recombinase polymerase amplification (RPA) [23,161], or PCR [162,163,164,165,166] have adopted this practice. 

However, side-by-side comparisons of nucleic acid testing on sputum samples with or without NaOH-NALC treatment have indicated that NaOH-NALC processing could be removed to enable truly direct protocols for sputum. Mitarai et al. reported that the addition of NALC to a sputum specimen prior to extraction had no effect on testing for TB [167]. Furthermore, Tarhan et al. showed that the sensitivity in TB testing was better for sputum samples that were measured directly, rather than after extraction with NaOH-NALC [168]. In pursuing alternatives to the lengthy NaOH-NALC method, sputum has been used in PCR after adding mucolytic agents [56,169], diluting in buffer [148], or bead-beating [157] to reduce viscosity. A more aggressive pretreatment, relying on chemical, thermal, and mechanical means of disruption, used heating and centrifugation to detect TB in 548 sputum samples with comparable performance to more expensive molecular-based systems [170]. One particularly noteworthy example of direct testing used LAMP to diagnose tuberculosis at three peripheral laboratories [147]. In the study, LAMP had a sensitivity of 97.9%, detecting 173 out of 177 smear-negative, culture-positive sputum samples. The authors canvassed the laboratory personnel after they implemented the heating, washing, and filter-tip capture steps before direct amplification to verify that the assay had significant potential to be adopted for routine use.

Several early examples of PCR on sputum samples with *Mycobacterium* spp. reported sensitivities in the single-digit copy number range. However, the pretreatment methods were quite divergent. Sjobring et al. used long centrifugation and sonication steps, in addition to boiling, to detect down to eight organisms [171]. Sritharan et al. was able to cut down the steps to a 30 min boiling period, with a resulting LOD of 1 organism [172]. Since these examples in the 1990s, only one technique with a pre-amplification wash and an RPA reaction has been able to match this performance in detecting a single mycobacterium [161]. As far as combining specificity and sensitivity without adding technical difficulty, Priye et al.’s recent multiplexed RT-LAMP detection system for Zika, dengue, and chikungunya achieved LODs of 44 copies/reaction with no need for lysis or extraction [82]. These impressive outcomes from isothermal technologies like RPA and LAMP illustrate that fancy hardware is not necessary for testing modalities to achieve high specificity directly in human samples.

### 4.6. Direct NAATs for Oral, Dermal, and Conjunctival Swabs

Swabs have become a mainstay in testing for viral pathogens. Molecular systems that identify respiratory tract infections in nasal swabs [13] or STIs (sexually transmitted infections) in dermal, genital, and conjunctival swabs [173] are used for rapid, accurate patient diagnosis. DNA collection from swabs is attractive because it is simple, minimally invasive, and even enables self-sampling. However, swab-collected specimens are likely to contain polymerase inhibitors such as secreted minerals, electrolytes, hormones, enzymes, immunoglobulins, and cytokines, as well as topical medications [137,174,175,176,177]. As a result, many swab tests now on the market remove these inhibitors via extraction methods that are too involved and complex to be suitable for the POC [178].

**Figure 7:**
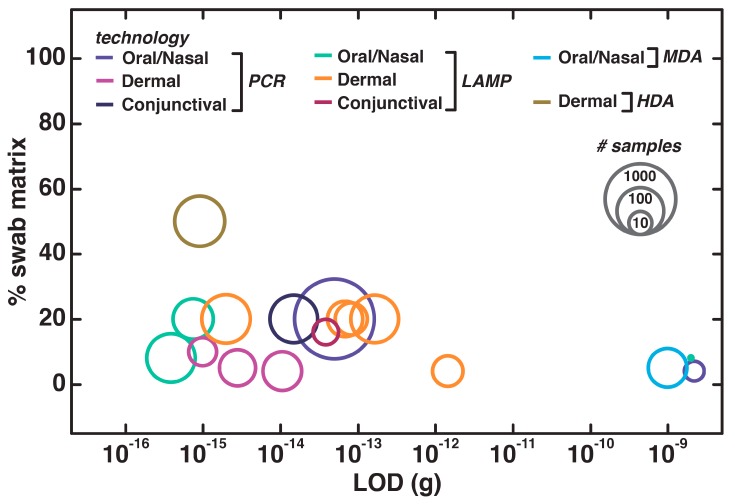
Swab-based procedures for nucleic acid testing, with data presented as in Figure 3.

All of the swab sample studies we examined employed at least one patient sample, and most achieved high sensitivity at a reasonable level of dilution in the buffer used for DNA elution from the solid swab (Figure 7). One remarkable study examined 4518 patient swabs in direct PCR for STR (short tandem repeat) analysis [179]—unfortunately, the authors did not report the yields of DNA obtained or the lowest amounts detected. This is a particularly common problem amongst these references—when the LODs are not reported, it is especially difficult to replicate these procedures or compare the manipulations used in sample storage, DNA replication, and detection [180,181]. Special attention should therefore be paid to reproducibility in future efforts at direct amplification of swab samples.

Typically, elution either at room temperature [51,176,182,183,184,185,186] or with heating [43,44,187,188,189,190,191] is sufficient to generate PCR-amplifiable template from swabs in solution. These methods of dilution or heating can save over thirty minutes of processing time, as with Nihonyanagi et al.’s heating protocol to release MRSA in CellEaseII (Biocosm Inc., Hyogo, Japan) diluents [169]. LAMP-based testing of swab samples can also be performed at room-temperature [192,193,194,195,196] or while heated [145,197,198,199,200,201,202], as can MDA [84]. In particular, Mahony et al.’s LAMP-based test for influenza A and B achieved an analytical sensitivity of one genome equivalent, operating via a novel swab preparation procedure of vortexing and heating [198].

Moving towards instrument-free molecular diagnostics systems, a lateral-flow strand-displacement amplification (SDA) [203] assay could directly detect MRSA from nasal swabs with a sensitivity of 600 copies/reaction [204]. Lateral flow eliminates the need for expensive detectors. Rogdriguez et al. went one step further by combining paper-based extraction and in situ amplification with lateral flow to develop an RT-LAMP assay for H1N1 in patient nasopharyngeal specimens. Their sensitivity of 500 copies/reaction was well below the mean viral load for H1N1 patients [205]. Pushing to even lower limits of detection, an HDA-based assay with a vertical-flow DNA strip readout [206] could directly test clinical genital swabs in transport medium for HSV types 1 and 2 [207]. The nucleic acid assays had LODs of 5.5 and 34.1 copies/reaction for HSV-1 and HSV-2, respectively, and were able to detect low-viral loads below the sensitivity of culture tests.

The most sensitive tests on swabs rely on LAMP reactions: for example, fewer than 10 copies/reaction of viral targets were seen after brief heating steps in Hank’s buffer (Whittaker Bioproducts, Boston, MA, USA) [197], M-Swab diluent (Copan Diagnostics Inc., Murrieta, CA, USA) [198], or water [202]. It is noteworthy is that entire swab samples can be used in amplification reactions, eliminating the loss of starting material that accompanies liquid and hardware transfers. This approach has also been successful in several instances of STR testing [43,179]. Rodriguez et al. used a similar approach in detecting clinical levels of H1N1 by passing an in-house elution buffer with the sample of interest through a filter paper-based setup, then amplifying on the filter membrane [205]. Future developments could focus on direct reactions with swabs that integrate extraction, amplification, and detection in a single tube for POC usage. This would give low-resource settings alternatives to instrument-dependent assays like the Alere i platform for detecting influenza A & B from nasal swabs.

### 4.7. Direct NAATs for Urine and Stool

Diseases of the kidney or genitourinary tract can often be detected from stool or urine. Urea destabilizes interactions between primers, template and polymerase, and since urea is typically present in adult urine at concentrations six-fold greater than can be tolerated in PCR reactions, ultracentrifugation, or related procedures are typically used to prepare such samples for nucleic acid testing [208,209,210]. Molecular tests for the identification of pathogens in stool rely on extraction methods to remove the proteinases, bile salts, polyphenols, and acids that directly inhibit the activity of DNA polymerases [211,212]. Methods for testing external specimens have been integrated into diagnostic screens for pathogens such as *Chlamydia trachomatis*, *Neisseria gonorrhoeae*, and *Clostridium difficile* with high sensitivity and specificity [213,214,215,216,217]. 

**Figure 8:**
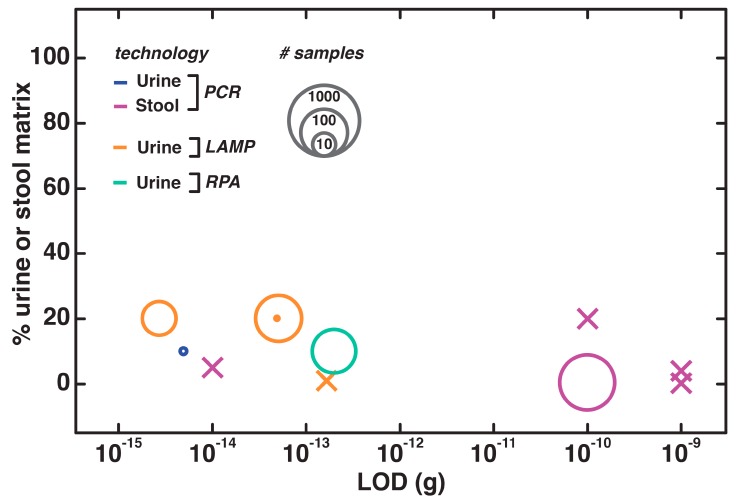
Nucleic acid testing in fecal and urine samples, with data presented as in Figure 3.

In surveying direct nucleic acid testing methods (Figure 8), we noted that the LODs are generally lower for urine-based tests than for feces-based. However, there is one example of direct PCR in stool samples, taking advantage of buffer additives and enhanced Phire polymerase (New England Biolabs, Ipswich, MA, USA), which achieved a LOD of 0.002 pg of bacterial DNA. However, there is a clear need for increased testing with patient samples, as nearly half of the referenced works do not include any clinical validation. This issue is especially notable with fecal testing, where patient testing is only reported in one study.

Though complex extraction methods are recommended prior to PCR in order to remove inhibitory components of urine and feces, changes to the reaction chemistry could effectively relieve the negative effects of the sample matrix. With urine, this alteration takes the form of a hydrogel-encased reaction [183]. The authors developed a pre-assembled, desiccated, gel-based cassette that is rehydrated by the liquid in raw urine samples. The polyacrylamide gel matrix effectively filters biological inhibitors out of the amplification reaction, enabling detection of *Mycoplasma homonis* and *Ureaplasma urealyticum*. For feces, the options are to employ inhibitor-resistant polymerases [57] and buffer additives [212]. In the case of one large-scale characterization by Hall et al., the combination of both Phire polymerase (New England Biolabs, Ipswich, MA, USA) and Ampdirect (Biomatrica, San Diego, CA) gave an LOD of nearly one copy/reaction in PCR for *Francisella tularensis* in 0.5% stool [56]. One can also lyse bacteria in urine [218] or stool [219,220] through heating to release an amplifiable amount of target DNA with minimal levels of inhibitors. Moore et al. managed to detect Human norovirus repeatedly in 11 out of 12 outbreak stool samples after boiling the diluted feces in PBS [221]. Although centrifugation is employed in some semi-direct methods to create a supernatant from the collected stool, we believe a dilution step could accomplish the same feat by allowing solids to settle at the base of a highly aqueous, non-viscous sample.

Isothermal amplification techniques like LAMP and RPA generally demonstrate a higher tolerance than PCR for urine, as they can be carried out directly. As such, LAMP-based assays without any pre-processing steps or chemical enhancements have detected the causative agents of viral infections [82,127,131] or STIs [222,223], and pathogenic bacteria such as *Escherichia coli* [224]. The developers of the recently established isothermal method SIBA showed the utility of this amplification technique by detecting *Chlamydia trachomatis* and *Neisseria gonorrhoeae* in a low-copy urine sample [25].

## 5. Discussion

The landscape of molecular diagnostics is constantly advancing, as is the current paradigm of healthcare. The advent of mobile health and telemedicine has decentralized patient care. It has also put a new emphasis on usability and non-invasiveness in disease testing. Nucleic acid diagnostics that reduce the difficulties and expenditures of standard multi-step procedures by direct amplification can expedite patient testing in POC, hospital, and laboratory situations [225]. In this review, we are thus motivated to discuss the current state of the art for direct NAATs: assays and platforms that require minimal or no sample preparation procedures. We search the literature from 1989 to 2017 and find 174 published works that we consider direct NAATs. We first categorize these works based on the type of complex samples. We subsequently employ bubble plots to facilitate the comparison of the amplification method, robustness in complex media, sensitivity to target, and clinical usage. Our findings indicate that the majority of direct NAATs exhibit a tolerance of less than 17% for their sample of interest, and fewer than 30 patient-based evaluations. Still, there are diagnostic procedures that far surpass these averages. Sim et al.’s study of direct PCR on 4518 buccal swabs [179], for instance, is robust and carried out directly on the entire sample for maximal ease. Improvements must continue to be made for all sample types in terms of facilitating this level of evaluation with clinical samples.

Despite significant developments to date, there remain several challenges for realizing direct NAATs with user-friendliness, consistency, and generalizability. In furthering the development of direct testing, it is important to take a holistic approach and consider the type of sample to be analyzed, the method of sample acquisition, the throughput and volume, and any chemical or mechanical requirements, and the amplification technique. Another key point to note is that different matrices will function best in different environments. An improved understanding of the mechanisms behind emerging nucleic acid amplification reactions and mutant polymerases will enable the rationalization of how inhibitory compounds can make or break an amplification system. Furthermore, molecular assays have much to learn from diagnostics that are continuously being developed in the commercial pipeline. Proprietary technology will always hold knowledge at a cost to the user, but the implementation of new ideas can lead researchers towards better and more successful ways in which to modernize the ever-changing field of disease testing.

## 6. Conclusions

As we enter the age of electronic, mobile, and personalized medicine, there remains much room for creativity and innovation in the design of NAATs and POC diagnostics. Molecular diagnostics, as the highest-growing segment of all in vitro diagnostic products [226], truly have great potential for both developed and low-resource areas. The diagnostic community continues to strive for tests that are reliable against variable electrical resources, water quality, trained staff, or harsh environmental conditions [227,228]. Researchers continue to seek approvals such as the FDA’s CLIA waivers or the WHO’s ASSURED (affordable, sensitive, specific, user-friendly, rapid and robust, equipment-free, and delivered to end-users) criteria. In this regard, direct NAATs present a promising approach. Through this review, it is our hope to stimulate the discussion on direct NAATs and their potential as POC diagnostics. Ultimately, we seek to help accelerate the development of POC diagnostics that can be CLIA waived and/or meet the WHO ASSURED criteria, thereby ushering in the next revolution in healthcare.

## Supplementary Materials

The following are available online at https://www.mdpi.com/2079-6374/9/4/117/s1, Table S1: Detailed Properties of Amplification Assays.
